# Supplementary material for: Blank Spectrum Correction as a Robust Solution to Artifacts in Quantitative X‑ray Fluorescence Mapping
Source: Anal Chem. 2026 Sep 16;98(38):28272–84. doi: 10.1021/acs.analchem.6c03597 (PMC13629300; doi:10.1021/acs.analchem.6c03597)
Supplement: Supplementary file 1 [file ac6c03597_si_001.pdf]

Supporting Information

Blank Spectrum Correction as a Robust Solution to Artifacts in Quantitative X-ray Fluorescence Mapping

Authors:

Andrew M. Crawford[,a], Julia Balough[b], Yu-Ying Chen[b], Qiaoling Jin[c], Keith W. MacRenaris[a], Seth Garwin[a], Teresa K. Woodruff[d,b], Chris Jacobsen[c], James E. Penner-Hahn[*,e], and Thomas V. O'Halloran[*,a,f]*

[a] Department of Microbiology, Genetics, and Immunology, Michigan State University, 567 Wilson Rd., East Lansing, Michigan 48824, United States

[b] Department of Obstetrics and Gynecology, Feinberg School of Medicine, Northwestern University, 303 E. Superior St., Chicago, Illinois 60611, United States

[c] Department of Physics and Astronomy, Northwestern University, 2145 Sheridan Rd., Evanston, Illinois 60208, United States

[d] Department of Obstetrics, Gynecology, and Reproductive Biology, Michigan State University, 965 Wilson Rd., East Lan-sing, Michigan 48824, United States

[e] Department of Chemistry, University of Michigan, 930N. University Ave., Ann Arbor, Michigan 48109, United States

[f] Department of Chemistry, Michigan State University, 578 S. Shaw Ln, East Lansing, MI, United States

[*] Corresponding Authors email: crawf472@msu.edu, jeph@umich.edu, ohallor8@msu.edu

Table of Contents

1. Table S1: The mean quantification and population distribution in units of μg/cm^2 from the ROI analysis of the 12 blastocysts shown in Fig. 1 ....S-4
2. Table S2: Relative percentage differences in quantification and population distribution from ROI analysis of the 12 blastocysts shown in Fig. 1, ...S-5
3. Figure S1: Pair-wise plots from paired-sample t-tests performed on the 12 blastocysts from Fig. 1 for chlorine, potassium, sulfur, and phosphorus................S-6
4. Figure S2: Pair-wise plots from paired-sample t-tests performed on the 12 blastocysts from Fig. 1 for calcium, zinc, iron, and copper....................................S-7
5. Figure S3: Pair-wise plots from paired-sample t-tests performed on the 12 blastocysts from Fig. 1 for cobalt, manganese, ambient argon, and substrate silicon. S-8
6. Figure S4: Comparison of blank spectra used in blank spectrum correction of the secondary follicle presented in Figure 2 of the main text S-9
7. Figure S5: Comparison of early-stage blastocysts...S-10
8. Figure S6: Comparison of 8-cell Embryo S-11
9. Figure S7: Comparison of Morula S-12
10. Figure S8: Spectra are of sufficient quality and counting statistics for quantification. S-13
11. Figure S9: Baseline distortion is systematically dependent on snip width used in the Python SNIP function. S-14
12. Figure S10: Tophat baseline distortion is variably dependent on peak separation in addition to dwell time. S-16
13. Figure S11: Boxcar baseline distortion is independent on peak separation and dwell time but results in consistent underestimation. S-17

1. **Table S1: The mean quantification and population distribution in units of μg/cm² from the ROI analysis of the 12 blastocysts shown in Fig. 1**

**Means**	*Si*	*P*	*S*	*Cl*	*Ar*	*K*	*Ca*	*Mn*	*Fe*	*Co*	*Cu*	*Zn*
Baseline (Aggregate)	-17.2	4.73	5.17	36.6	-0.137	9.89	0.274	3.63×10^{-3}	1.25×10^{-2}	1.64×10^{-4}	3.79×10^{-3}	5.98×10^{-2}
Baseline (Per-Pixel)	-13.6	7.59	7.09	41.4	-0.131	10.8	0.313	7.37×10^{-3}	1.49×10^{-2}	2.53×10^{-3}	5.34×10^{-3}	6.06×10^{-2}
Blank (Aggregate)	-3.01	2.48	2.99	19.6	0.0407	7.09	0.241	-1.81×10^{-4}	1.39×10^{-2}	5.04×10^{-4}	4.36×10^{-3}	6.64×10^{-2}
Blank (Per-Pixel)	-3.01	2.48	2.99	19.6	0.0407	7.09	0.241	-1.81×10^{-4}	1.39×10^{-2}	5.04×10^{-4}	4.36×10^{-3}	6.64×10^{-2}
**Standard Deviations**	*Si*	*P*	*S*	*Cl*	*Ar*	*K*	*Ca*	*Mn*	*Fe*	*Co*	*Cu*	*Zn*
Baseline (Aggregate)	5.05	0.988	0.822	7.10	3.28×10^{-2}	2.34	8.98×10^{-2}	1.10×10^{-3}	4.37×10^{-3}	1.33×10^{-4}	1.07×10^{-3}	1.53×10^{-2}
Baseline (Per-Pixel)	5.85	1.27	1.07	7.56	3.92×10^{-2}	2.70	9.35×10^{-2}	1.96×10^{-3}	5.08×10^{-3}	6.31×10^{-4}	1.35×10^{-3}	1.65×10^{-2}
Blank (Aggregate)	1.18	0.403	0.418	3.39	2.28×10^{-2}	1.64	7.05×10^{-2}	3.88×10^{-4}	4.62×10^{-3}	9.81×10^{-5}	1.14×10^{-3}	1.68×10^{-2}
Blank (Per-Pixel)	1.18	0.403	0.418	3.39	2.28×10^{-2}	1.64	7.05×10^{-2}	3.88×10^{-4}	4.62×10^{-3}	9.81×10^{-5}	1.14×10^{-3}	1.68×10^{-2}

Table S1: The mean quantification and population distribution in units of μg/cm² from the ROI analysis of the 12 blastocysts shown in Fig. 1 resulting from baseline subtraction and blank spectrum correction of per-pixel and aggregate spectra. Comparison of blank spectrum correction for aggregate and per-pixel spectra shows the answers are identical and therefore independent of any degree of signal aggregation.

2. **Table S2: Relative percentage differences in quantification and population distribution from ROI analysis of the 12 blastocysts shown in Fig. 1,**

	Blank (Aggregate vs. Per-Pixel)			
	**Δ Conc.**	**Δ Pop. Dist.**	**Paired T-Test**	**Unpaired T-Test**
Si	-3.9×10^{-11} %	3.21×10^{-11} %	#DIV/0	1
P	-2.56×10^{-10} %	-5.23×10^{-10} %	#DIV/0	1
S	-5.56×10^{-11} %	-5.65×10^{-10} %	0.999146	1
Cl	1.82×10^{-10} %	4.62×10^{-10} %	#DIV/0	1
Ar	2.4×10^{-11} %	-7.58×10^{-11} %	0.99992	1
K	-1.14×10^{-10} %	6.15×10^{-10} %	#DIV/0	1
Ca	-1.78×10^{-10} %	-1.58×10^{-10} %	#DIV/0	1
Mn	-1.05×10^{-9} %	-5.43×10^{-11} %	0.9999	1
Fe	-1.82×10^{-10} %	-1.10×10^{-10} %	#DIV/0	1
Co	2.14×10^{-10} %	-1.37×10^{-10} %	#DIV/0	1
Cu	7.85×10^{-11} %	7.33×10^{-11} %	0.999551	1
Zn	9.45×10^{-11} %	1.53×10^{-10} %	0.999273	1

Table S2: Relative percentage differences in quantification and population distribution from ROI analysis of the 12 blastocysts shown in Fig. 1, and tabulated in Table S1, resulting from blank spectrum correction of per-pixel and aggregate spectra. Statistical differences were determined using a paired-sample t-test, with the results of unpaired-sample t-test shown as a courtesy.

3. Figure S1: Pair-wise plots from paired-sample t-tests performed on the 12 blastocysts from Fig. 1 for chlorine, potassium, sulfur, and phosphorus.

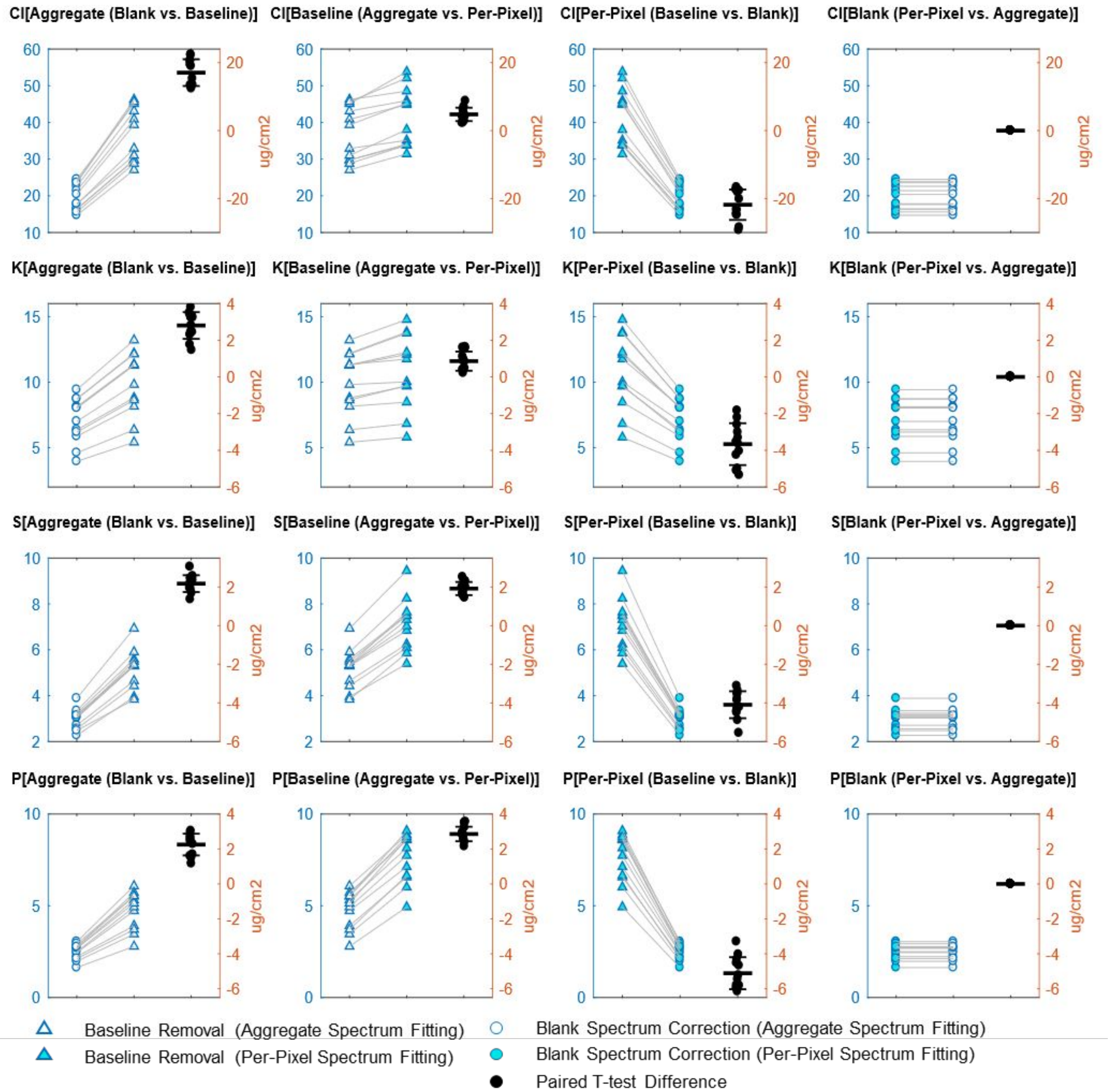

Figure S2: Pair-wise plots from paired-sample t-tests performed on the 12 blastocysts from Fig. 1 comparing the quantitative analysis between baseline and blank spectrum correction of per-pixel and aggregate spectra for chlorine, potassium, sulfur, and phosphorus. The left side of each plot shows the per-blastocyst change in quantitative estimates from the corresponding data reduction approach. The right side of each plot shows the absolute change in quantitative estimate for each pair.

4. **Figure S2: Pair-wise plots from paired-sample t-tests performed on the 12 blastocysts from Fig. 1 for calcium, zinc, iron, and copper.**

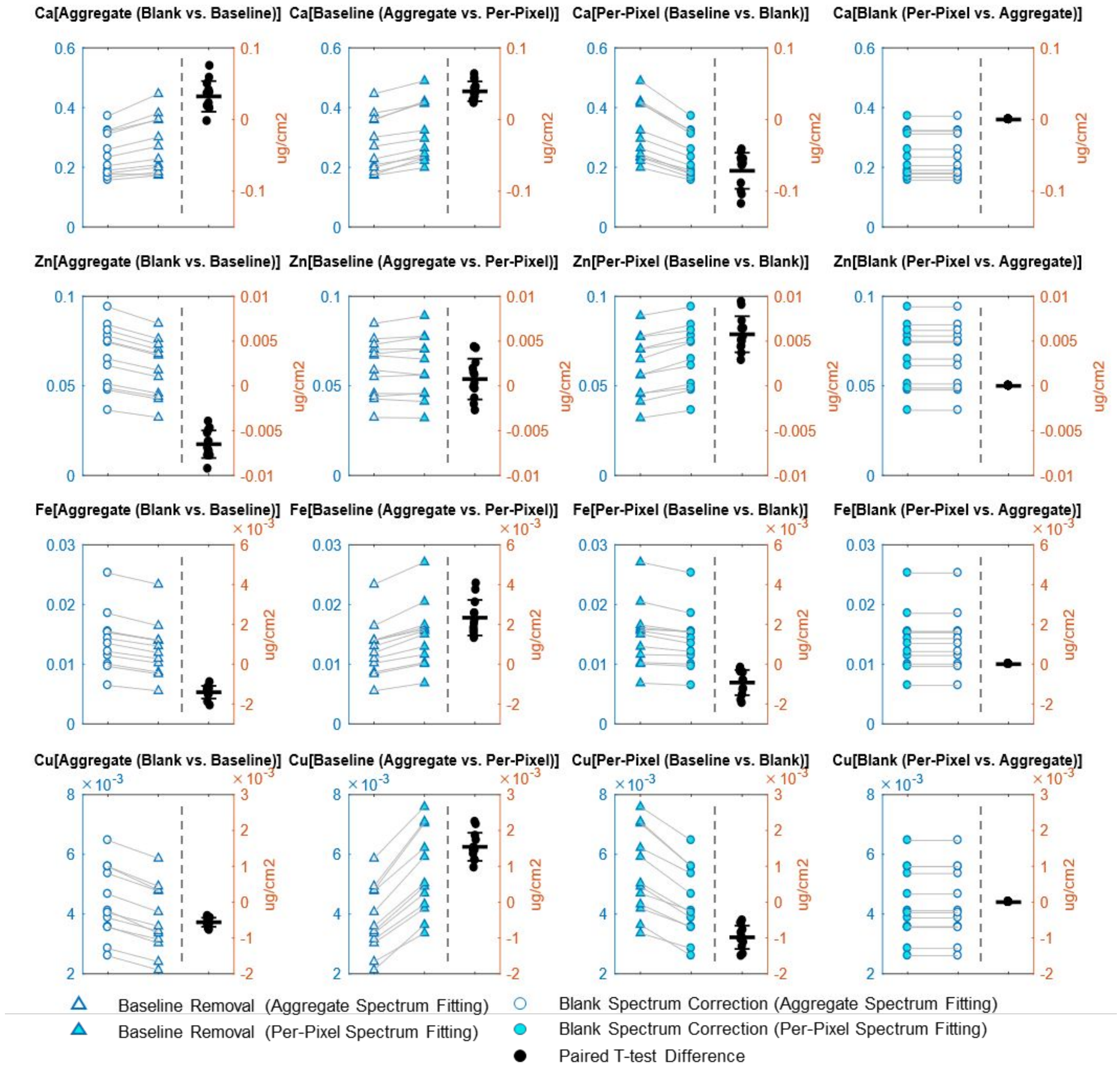

Figure S3: Pair-wise plots from paired-sample t-tests performed on the 12 blastocysts from Fig. 1 comparing the quantitative analysis between baseline and blank spectrum correction of per-pixel and aggregate spectra for calcium, zinc, iron, and copper. The left side of each plot shows the per-blastocyst change in quantitative estimates from the corresponding data reduction approach. The right side of each plot shows the absolute change in quantitative estimate for each pair.

5. Figure S3: Pair-wise plots from paired-sample t-tests performed on the 12 blastocysts from Fig. 1 for cobalt, manganese, ambient argon, and substrate silicon.

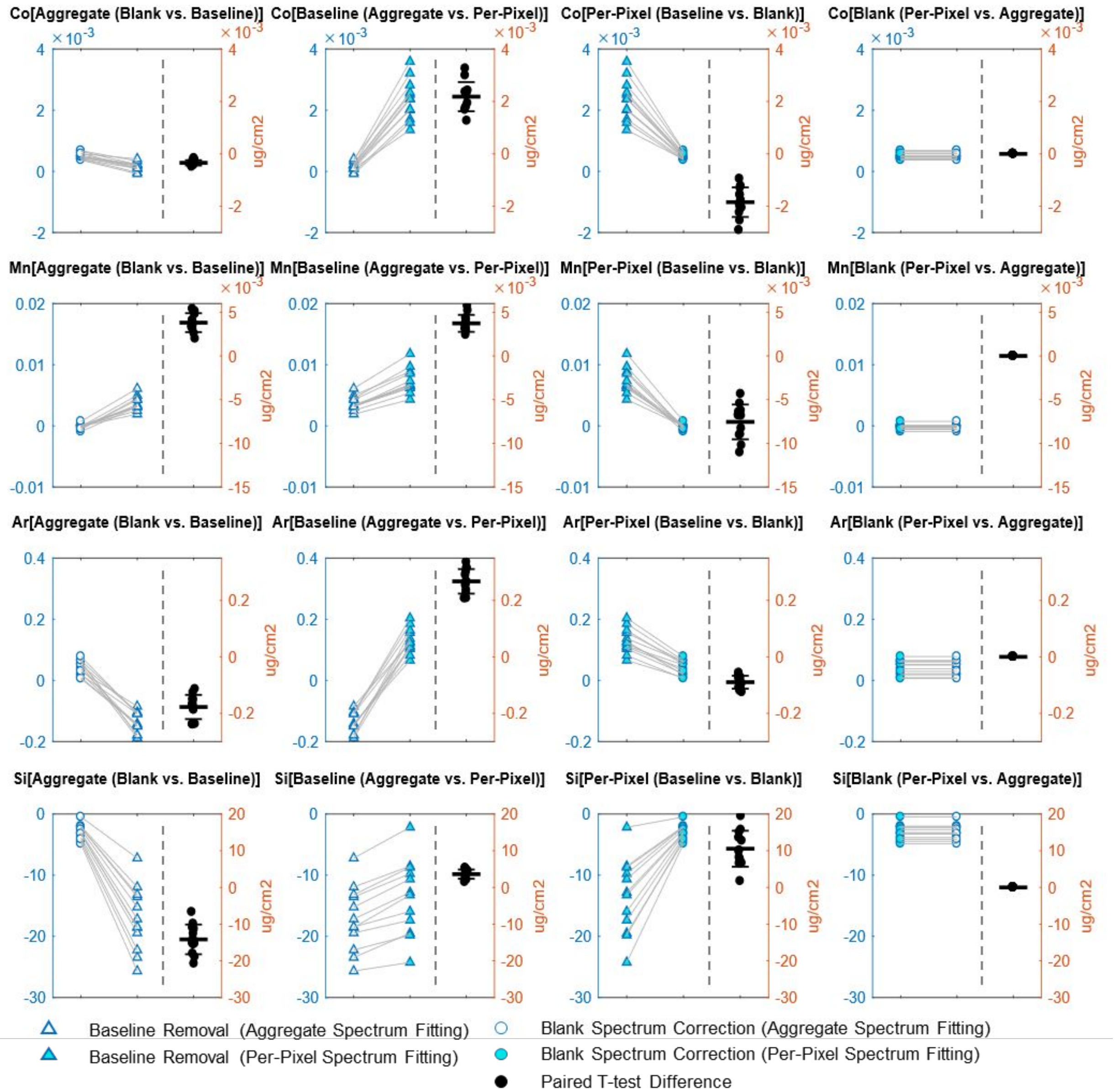

Figure S4: Pair-wise plots from paired-sample t-tests performed on the 12 blastocysts from Fig. 1 comparing the quantitative analysis between baseline and blank spectrum correction of per-pixel and aggregate spectra for cobalt, manganese, ambient argon, and substrate silicon. The left side of each plot shows the per-blastocyst change in quantitative estimates from the corresponding data reduction approach. The right side of each plot shows the absolute change in quantitative estimate for each pair.

6. **Figure S4: Comparison of blank spectra used in blank spectrum correction of the secondary follicle presented in Figure 2 of the main text**

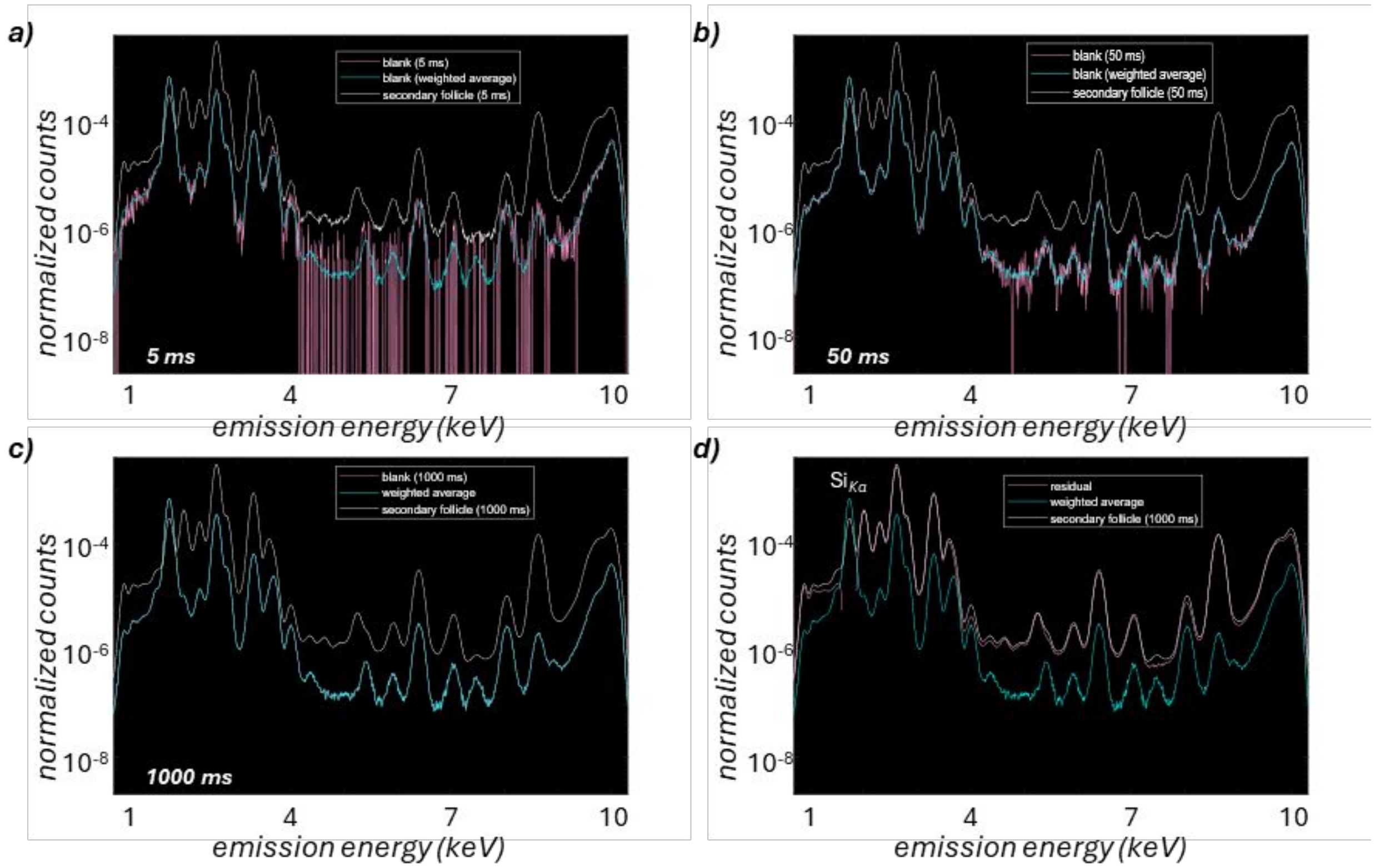

Figure S4: **Comparison of blank spectra used in blank spectrum correction of the secondary follicle presented in Figure 2 of the main text**. X-ray fluorescence microscopy mapping of a subregion of a murine secondary ovarian follicle was performed at dwell times of 5 ms, 50 ms, and 1000 ms with subsequent background correction performed in M-BLANK using the blank spectrum correction approach. (a-c) show the calculated blanks from the different dwell times of 5 ms, 50 ms, and 1000 ms are shown along with the blank obtained from the weighted average of all three. d) shows the residual signal post blank spectrum correction of the 1000 ms dwell time image corrected using the weighted average blank.

7. Figure S5: Comparison of early-stage blastocysts

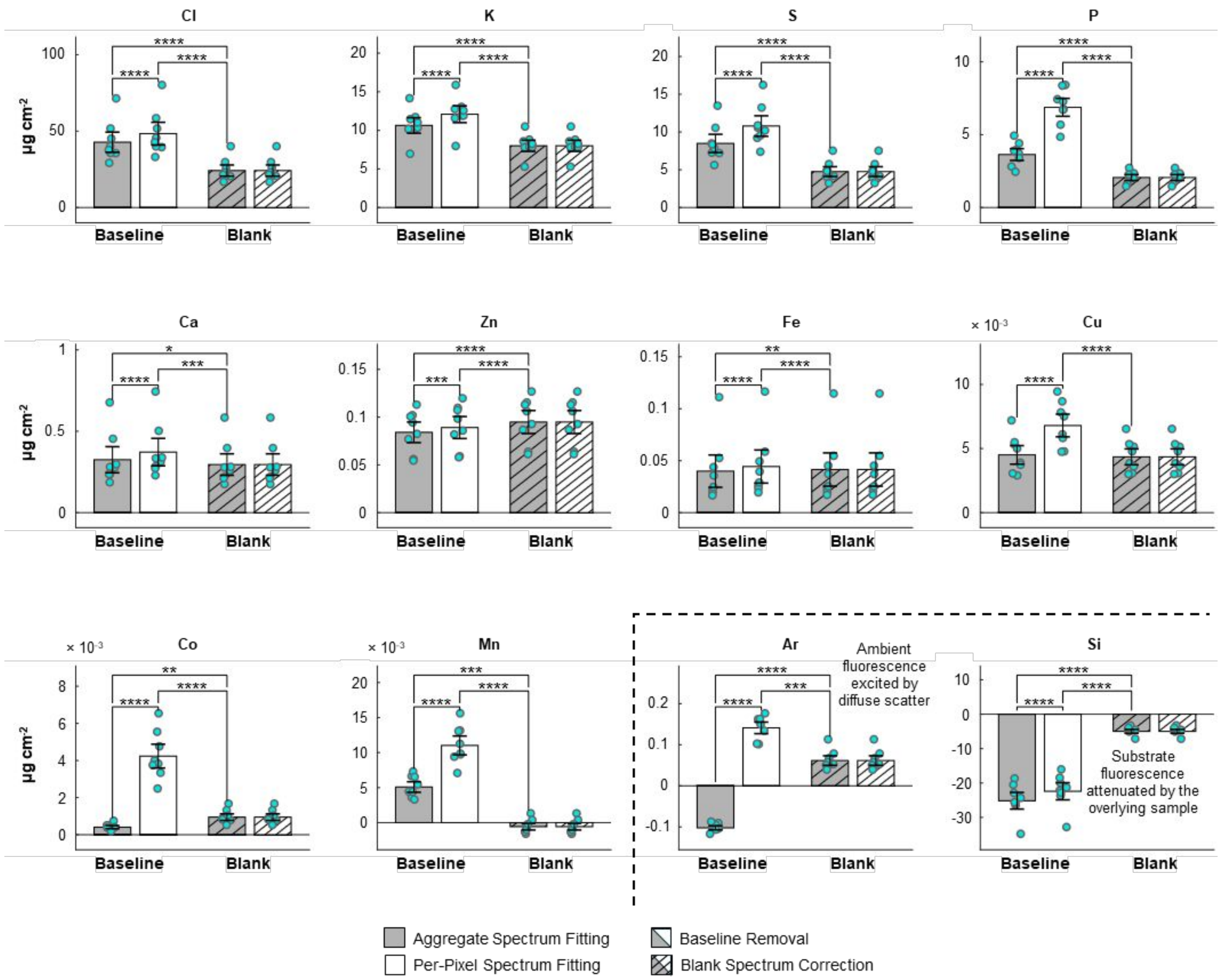

Figure S5: Comparison of early-stage blastocysts - Differences in quantitative analysis of early-stage blastocysts between baseline and blank spectrum correction of per-pixel and aggregate spectra. When analyzing the concentration of relevant elements across a set of 8 early stage blastocytes, significant quantitative differences exist depending on whether baseline or blank background subtraction methods were used and whether analysis was performed on individual pixel spectra or the aggregate spectrum of image ROIs. Quantitatively, blank spectrum correction fitting of per-pixel and aggregate spectra differed negligibly with relative root-mean-squared-errors ca. 1×10^{-14}. However, baseline removal of per-pixel and aggregate spectra never agreed, nor did either agree with quantitation from blank spectrum correction fitting. Bar plots correspond to the population mean. Errors bars are set at one standard deviation. Statistical differences were determined using a paired-sample t-test: * $p<0.05$; ** $p<0.01$; *** $p<0.001$; **** $p<0.0001$.

8. Figure S6: Comparison of 8-cell Embryo

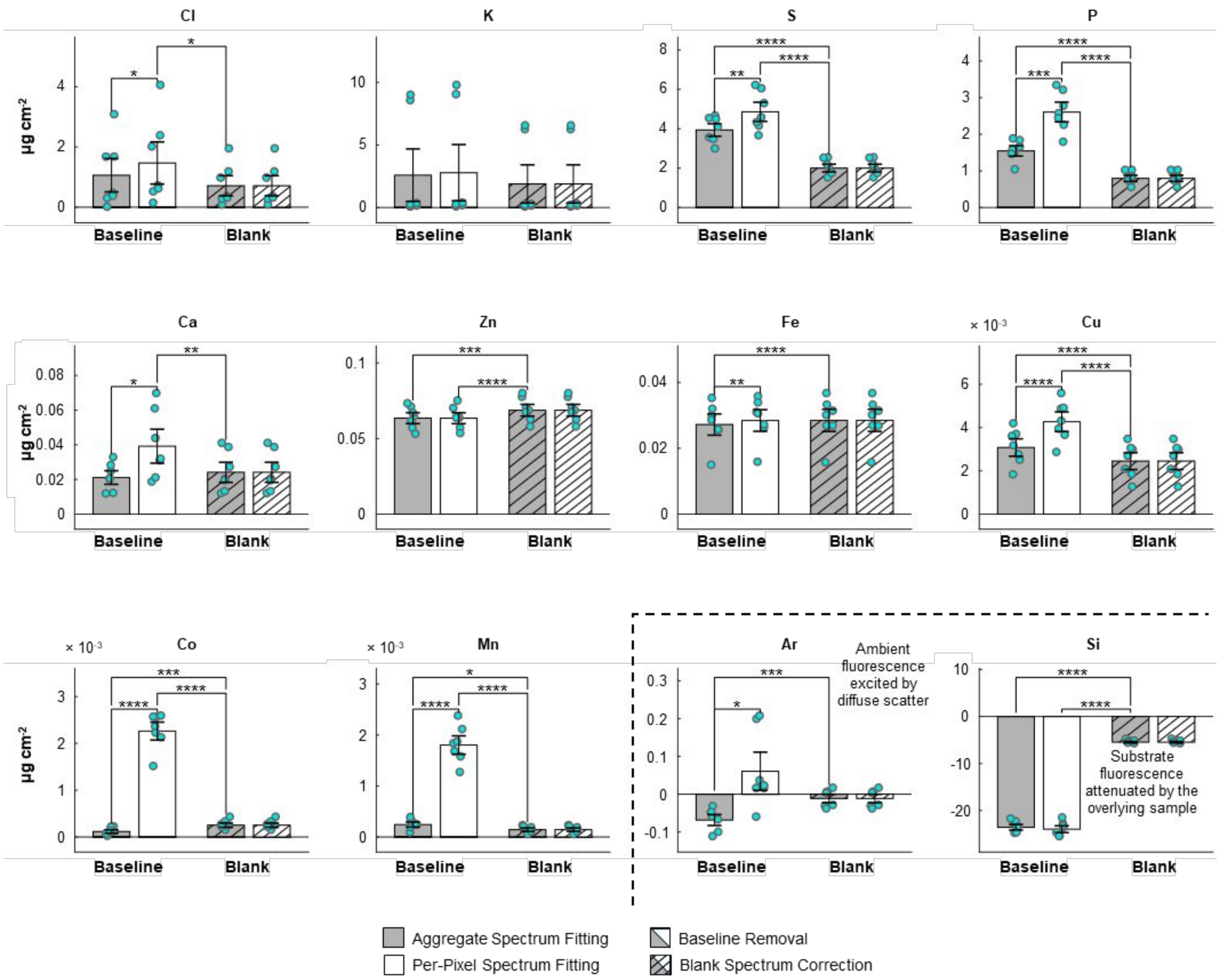

Figure S6: Comparison of 8-cell Embryo - Differences in quantitative analysis of 8-cell stage embryos between baseline and blank spectrum correction of per-pixel and aggregate spectra. When analyzing the concentration of relevant elements across a set of 7 8-cell stage embryos, significant quantitative differences exist depending on whether baseline or blank background subtraction methods were used and whether analysis was performed on individual pixel spectra or the aggregate spectrum of image ROIs. Quantitatively, blank spectrum correction fitting of per-pixel and aggregate spectra differed negligibly with relative root-mean-squared-errors ca. 1×10^{-14}. However, baseline removal of per-pixel and aggregate spectra never agreed, nor did either agree with quantitation from blank spectrum correction fitting. Bar plots correspond to the population mean. Errors bars are set at one standard deviation. Statistical differences were determined using a paired-sample t-test: * $p<0.05$; ** $p<0.01$; *** $p<0.001$; **** $p<0.0001$.

9. Figure S7: Comparison of Morula

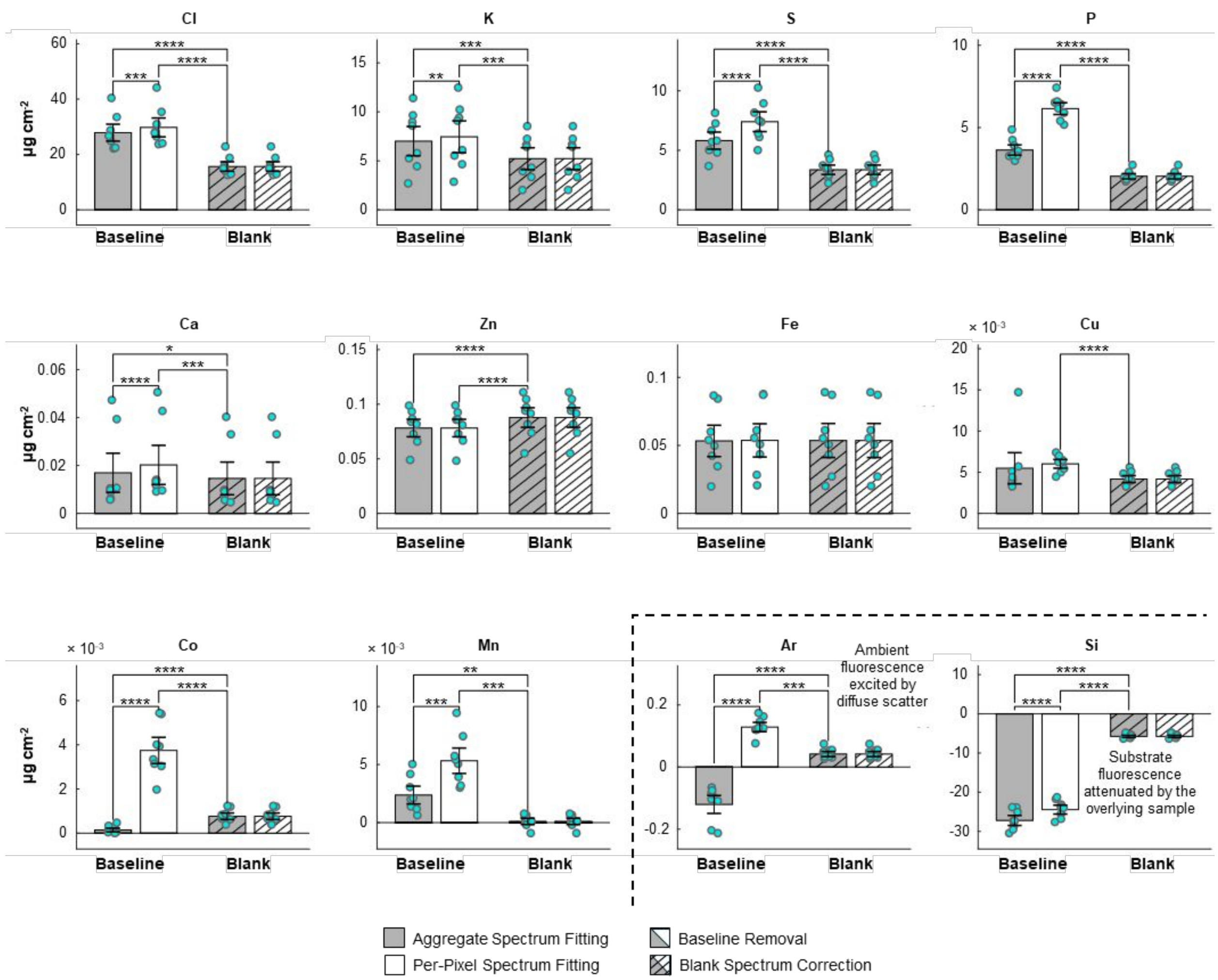

Figure S7: Comparison of Morula - Differences in quantitative analysis of 8 morula stage embryos between baseline and blank spectrum correction of per-pixel and aggregate spectra. When analyzing the concentration of relevant elements across a set of 7 8-cell stage embryos, significant quantitative differences exist depending on whether baseline or blank background subtraction methods were used and whether analysis was performed on individual pixel spectra or the aggregate spectrum of image ROIs. Quantitatively, blank spectrum correction fitting of per-pixel and aggregate spectra differed negligibly with relative root-mean-squared-errors ca. 1×10^{-14}. However, baseline removal of per-pixel and aggregate spectra never agreed, nor did either agree with quantitation from blank spectrum correction fitting. Bar plots correspond to the population mean. Errors bars are set at one standard deviation. Statistical differences were determined using a paired-sample t-test: * $p<0.05$; ** $p<0.01$; *** $p<0.001$; **** $p<0.0001$.

10. Figure S8: Spectra are of sufficient quality and counting statistics for quantification.

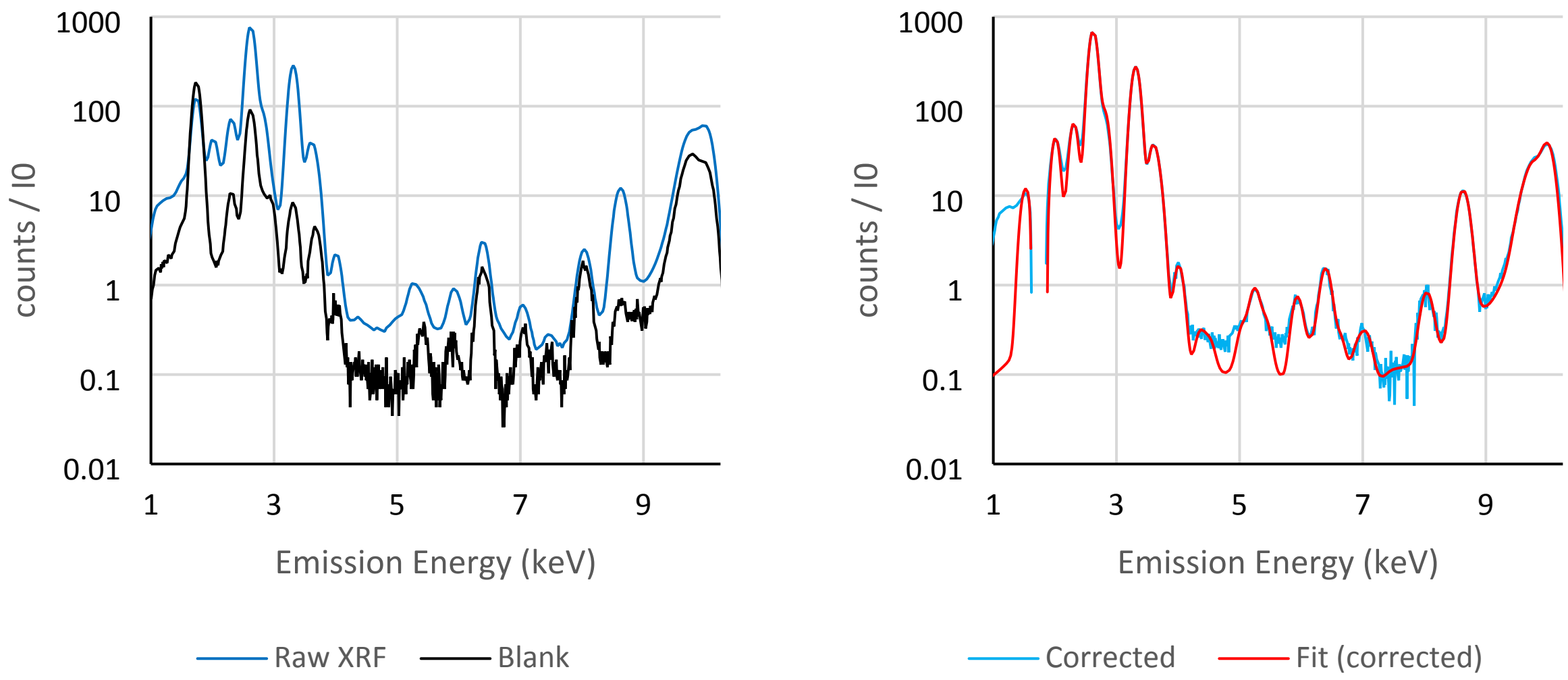

Figure S8: Spectra are of sufficient quality and counting statistics for quantification. The above show (left) the raw XRF along with the blank spectrum, and (right) the blank corrected spectrum and associated fit, for the blastocyst displayed in Fig. 1 of the manuscript. This is one of twelve blastocysts plotted. The fits were performed per-pixel and the resulting individual fits integrated combined.

11. Figure S9: Baseline distortion is systematically dependent on snip width used in the Python SNIP function.

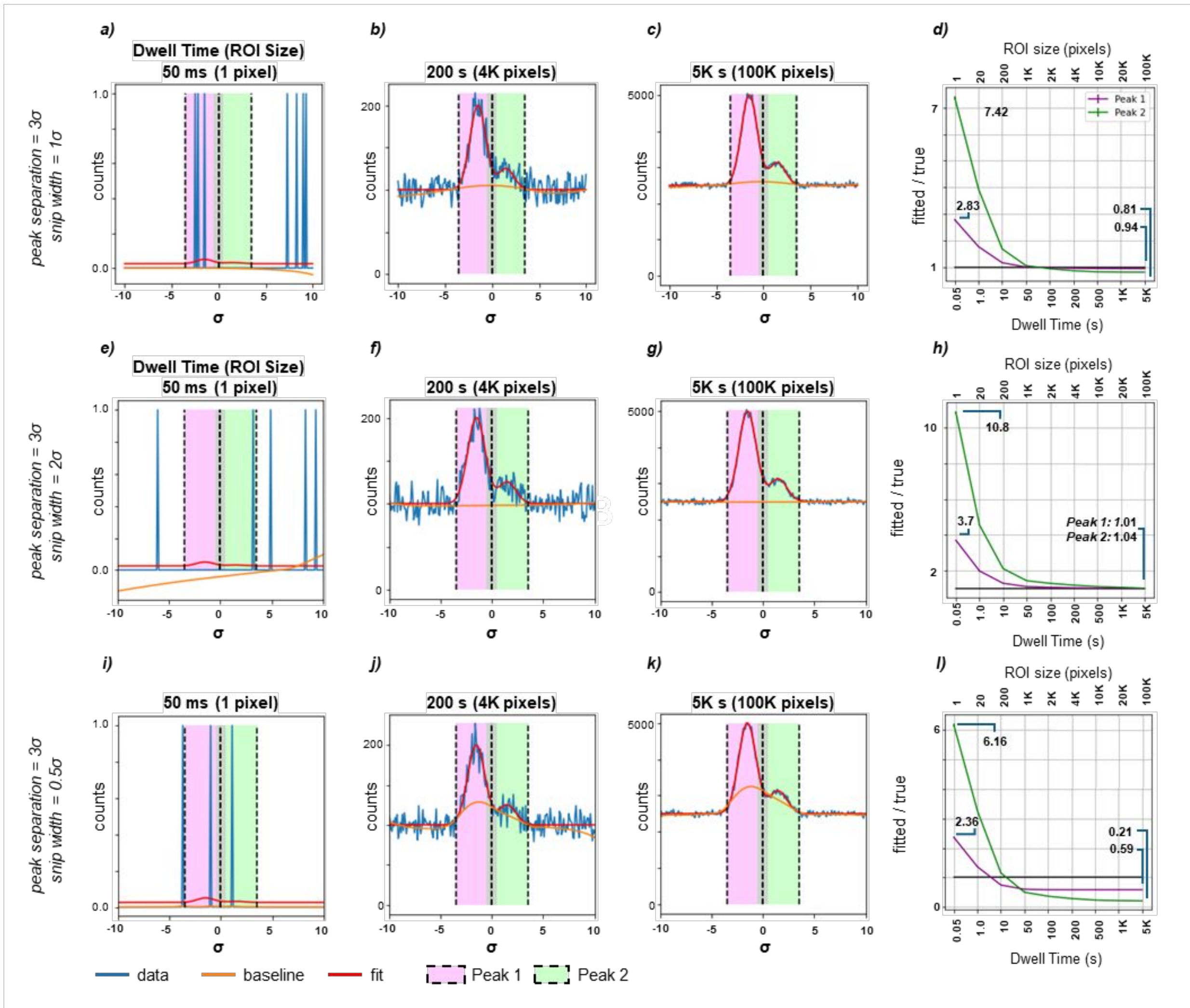

Figure S9: Baseline distortion is systematically dependent on snip width used in the Python SNIP function. We used a simple model consisting of two Gaussian functions riding on a constant linear background to explore the apparent peak area as a function of statistical noise, calculated from a Poisson distribution. The model was defined to have a signal-to-noise ratio of 1 for 1 second. We started with a single Gaussian with peak height of 1 and a background of 1. To study the effect of peak overlap, a second, smaller peak (peak height = 0.25) was added. Both Gaussians had a full-width half-maximum of 1 and were separated by 3 standard deviation (σ) units. Simulations were done for dwell times ranging from 50 milliseconds to 5,000 seconds (effective image ROI sizes of 1 pixel to 100,000 pixels), with representative simulations for 50 milliseconds (a, e, and i), 200 seconds (b, f, and j), and 5,000 seconds (c, g, and k) shown, with subsequent comparisons of changes in quantitative peak areas (d, h, and i). For each of the resulting data sets, baseline subtraction was modeled as a SNIP baseline calculated using the

pybaselines implementation of the SNIP algorithm (https://pypi.org/project/pybaselines/) with 5 point moving-average smooth (keyword smooth_half_window = 2). The effect of varying the snip-width (expected full-width-at-half-max; keyword max_half_window = σ) was tested for σ = 1 (a-c), 2 (e-g), and 0.5 (i-k). For each simulation, the ratio of fitted counts to the true counts was determined. To estimate reliability of the simulations, each condition was repeated 10,000 times and the resulting ratios were averaged to give the mean and standard deviation.

12. Figure S10: Tophat baseline distortion is variably dependent on peak separation in addition to dwell time.

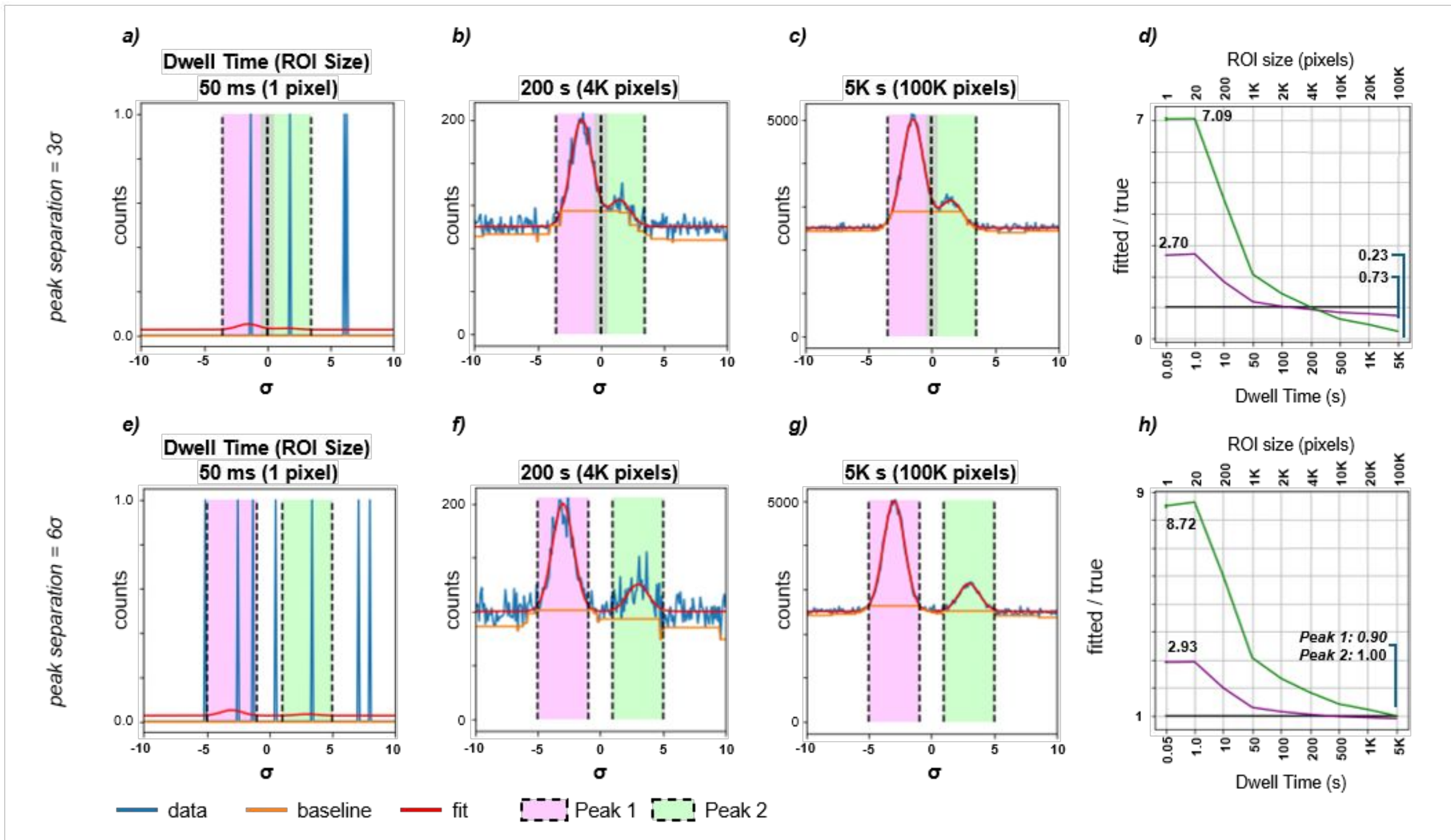

Figure S10: Tophat baseline distortion is variably dependent on peak separation in addition to dwell time. We used a simple model consisting of two Gaussian functions riding on a constant linear background to explore the apparent peak area as a function of statistical noise, calculated from a Poisson distribution. The model was defined to have a signal-to-noise ratio of 1 for 1 second. We started with a single Gaussian with peak height of 1 and a background of 1. To study the effect of peak overlap, a second, smaller peak (peak height = 0.25) was added. Both Gaussians had a full-width half-maximum of 1 and were separated by 3 (a-d) and 6 (e-h) standard deviation units. Simulations were done for dwell times ranging from 50 milliseconds to 5,000 seconds (effective image ROI sizes of 1 pixel to 100,000 pixels), with representative simulations for 50 milliseconds (a and e), 200 seconds (b and f), and 5,000 seconds (c and g) shown, with subsequent comparisons of changes in quantitative peak areas (d and h). For each simulated spectrum, the baseline was estimated using a digital top-hat approach, in which a morphological opening was used to approximate the local background before subtraction and peak-area integration. For each simulation, the ratio of fitted counts to the true counts was determined. To estimate reliability of the simulations, each condition was repeated 10,000 times and the resulting ratios were averaged to give the mean and standard deviation.

13. **Figure S11: Boxcar baseline distortion is independent on peak separation and dwell time but results in consistent underestimation.**

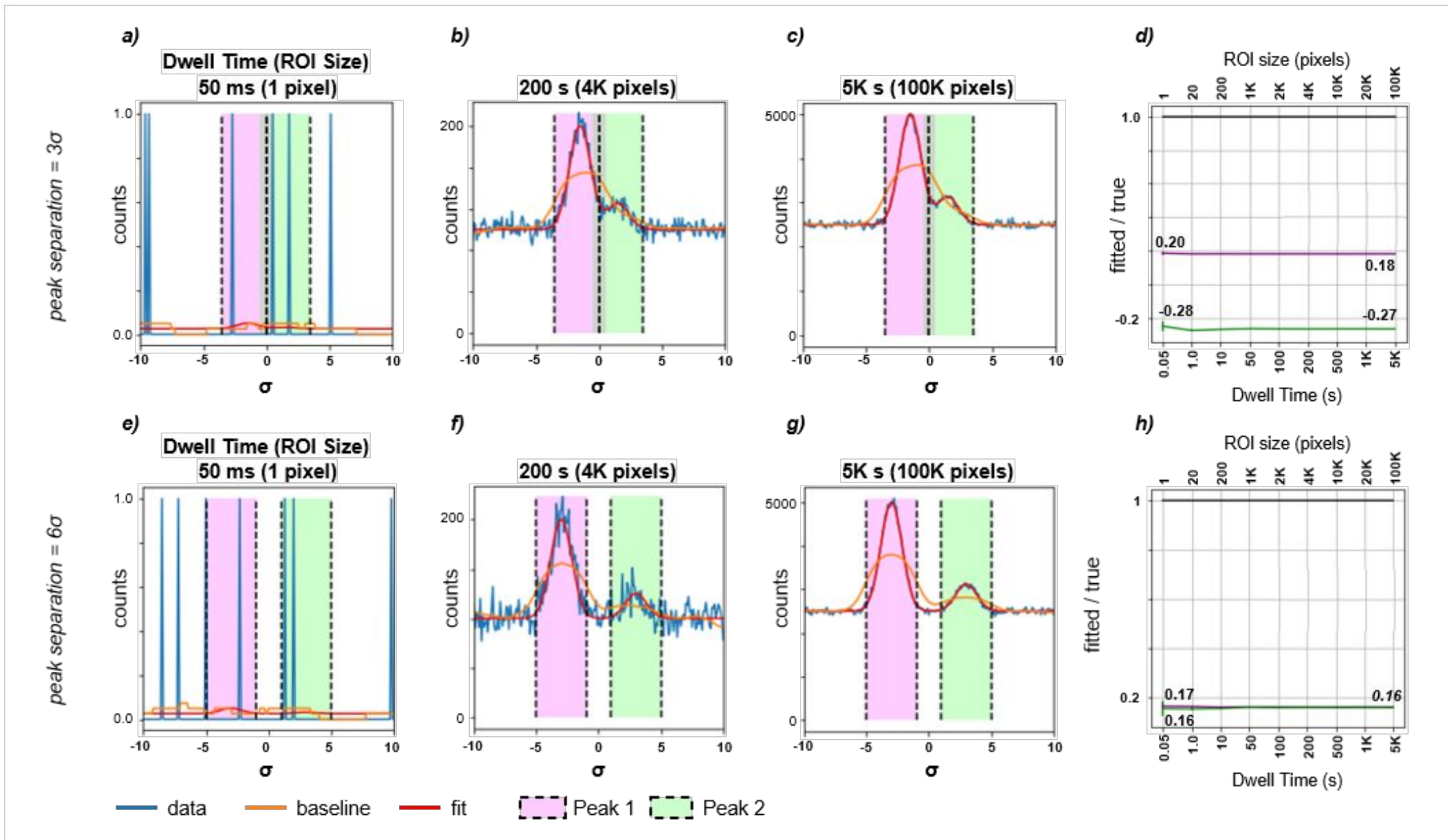

Figure S11: Boxcar baseline distortion is independent on peak separation and dwell time but results in consistent underestimation. We used a simple model consisting of two Gaussian functions riding on a constant linear background to explore the apparent peak area as a function of statistical noise, calculated from a Poisson distribution. The model was defined to have a signal-to-noise ratio of 1 for 1 second. We started with a single Gaussian with peak height of 1 and a background of 1. To study the effect of peak overlap, a second, smaller peak (peak height = 0.25) was added. Both Gaussians had a full-width half-maximum of 1 and were separated by 3 (a-d) and 6 (e-h) standard deviation units. Simulations were done for dwell times ranging from 50 milliseconds to 5,000 seconds (effective image ROI sizes of 1 pixel to 100,000 pixels), with representative simulations for 50 milliseconds (a and e), 200 seconds (b and f), and 5,000 seconds (c and g) shown, with subsequent comparisons of changes in quantitative peak areas (d and h). For each simulated spectrum, the baseline was estimated by applying a boxcar smoothing filter to the noisy spectrum prior to baseline subtraction and peak-area integration. For each simulation, the ratio of fitted counts to the true counts was determined. To estimate reliability of the simulations, each condition was repeated 10,000 times and the resulting ratios were averaged to give the mean and standard deviation.
